# Exploring Shared Implementation Leadership of Point of Care Nursing Leadership Teams on Inpatient Hospital Units: Protocol for a Collective Case Study

**DOI:** 10.2196/54681

**Published:** 2024-02-19

**Authors:** Sonia Angela Castiglione, Mélanie Lavoie-Tremblay, Kelley Kilpatrick, Wendy Gifford, Sonia Elizabeth Semenic

**Affiliations:** 1 Ingram School of Nursing McGill University Montreal, QC Canada; 2 Faculty of Nursing Université de Montréal Montreal, QC Canada; 3 Faculty of Health Sciences University of Ottawa Ottawa, ON Canada

**Keywords:** case study, evidence-based practices, implementation leadership, inpatient hospital units, nursing leadership, point of care

## Abstract

**Background:**

Nursing leadership teams at the point of care (POC), consisting of both formal and informal leaders, are regularly called upon to support the implementation of evidence-based practices (EBPs) in hospital units. However, current conceptualizations of effective leadership for successful implementation typically focus on the behaviors of individual leaders in managerial roles. Little is known about how multiple nursing leaders in formal and informal roles share implementation leadership (IL), representing an important knowledge gap.

**Objective:**

This study aims to explore shared IL among formal and informal nursing leaders in inpatient hospital units. The central research question is as follows: How is IL shared among members of POC nursing leadership teams on inpatient hospital units? The subquestions are as follows: (1) What IL behaviors are enacted and shared by formal and informal leaders? (2) What social processes enable shared IL by formal and informal leaders? and (3) What factors influence shared IL in nursing leadership teams?

**Methods:**

We will use a collective case study approach to describe and generate an in-depth understanding of shared IL in nursing. We will select nursing leadership teams on 2 inpatient hospital units that have successfully implemented an EBP as instrumental cases. We will construct data through focus groups and individual interviews with key informants (leaders, unit staff, and senior nurse leaders), review of organizational documents, and researcher-generated field notes. We have developed a conceptual framework of shared IL to guide data analysis, which describes effective IL behaviors, formal and informal nursing leaders’ roles at the POC, and social processes generating shared leadership and influencing contextual factors. We will use the Framework Method to systematically generate data matrices from deductive and inductive thematic analysis of each case. We will then generate assertions about shared IL following a cross-case analysis.

**Results:**

The study protocol received research ethics approval (2022-8408) on February 24, 2022. Data collection began in June 2022, and we have recruited 2 inpatient hospital units and 25 participants. Data collection was completed in December 2023, and data analysis is ongoing. We anticipate findings to be published in a peer-reviewed journal by late 2024.

**Conclusions:**

The anticipated results will shed light on how multiple and diverse members of the POC nursing leadership team enact and share IL. This study addresses calls to advance knowledge in promoting effective implementation of EBPs to ensure high-quality health care delivery by further developing the concept of shared IL in a nursing context. We will identify strategies to strengthen shared IL in nursing leadership teams at the POC, informing future intervention studies.

**International Registered Report Identifier (IRRID):**

DERR1-10.2196/54681

## Introduction

### Overview

Leadership is a key factor in generating conducive contexts for the successful implementation of evidence-based practices (EBPs) in health care settings [1]. Developing effective point of care (POC) nursing leadership for implementation, or implementation leadership (IL), has the potential to create optimal climates for implementation and improve patient outcomes [2]. However, the current understanding of IL in nursing is limited and does not consider the diversity of formal and informal leadership roles that are involved in implementation [3]. In addition, formal and informal nurse leaders work within nursing leadership teams to lead implementation efforts, yet how nursing leaders share in this process has not yet been described. The exploration of IL in nursing is important since it is well known that successful implementation of EBPs in nursing continues to be elusive, contributing to persistent quality gaps in care delivery. Furthermore, within the existing culture of continuous and relentless change, failed implementation efforts may intensify nurses’ experiences of physical and emotional sequelae of “change fatigue” including exhaustion, apathy, powerlessness, and burnout, which in turn threaten their engagement in further change initiatives [4].

This study will explore and deepen a relational view of IL, called shared IL, which resonates with the number and variety of nursing roles involved in implementation at the POC. Specifically, this study will describe the leadership behaviors of nurses in formal managerial and nonmanagerial leadership roles at the POC, including nurse managers (NMs), assistant nurse managers (ANMs), nursing professional development educators (NPDEs), and advanced practice nurses (APNs), as well as informal leaders such as champions in the change process who are often appointed to support local implementation efforts. In addition, this study will explore the social processes where shared IL emerges among the various leaders. Finally, the contextual factors that enable shared IL will be highlighted. A more holistic understanding of shared IL in nursing can support the development of effective leadership for implementation.

### Background

IL is a nascent concept in implementation science literature that provides guidance on what POC leadership behaviors need to be developed and performed to support successful implementation [5]. IL describes strategic leadership behaviors based on transformational and effective leadership behavior theories whereby leaders are visibly committed, supportive of POC staff, and engaged at the granular level to prepare for and facilitate implementation [6,7]. These behaviors are posited to strengthen implementation climates, which consist of “employees’ shared perceptions of the policies, practices, procedures and behaviors that are rewarded, supported and expected in order to facilitate effective EBP implementation and use” [8]. Recent evidence from a nonnursing longitudinal study in mental health clinics supports a link between the enactment of IL behaviors by POC clinical managers with higher levels of implementation climate and self-reported adoption of EBPs by clinicians, reinforcing the critical role of POC leaders in creating the conditions for implementation to succeed [9].

IL has been typically conceptualized as an approach that is manifested through the behaviors of individual leaders, in particular leaders who hold managerial positions at the POC [3]. Indeed, a growing body of research describes NMs as playing a vital role contributing to implementation success. In an updated systematic review, Gifford et al [10] found that NMs enacted change-, relational-, and task-oriented behaviors that included communicating organizational priorities for change, establishing partnerships with nursing and interprofessional colleagues, participating in planning for implementation, and providing resources. These behaviors indicated to staff that the use of the EBP in clinical practice was important and served to “inspire, encourage and provide tangible incentives to staff” to adopt EBPs [10]. Birken et al [11] similarly found that middle managers (including POC NMs) played multiple roles, which consisted of encouraging and enabling frontline staff to overcome obstacles, addressing concerns, coaching, and providing incentives to shape an implementation-conducive climate. These reviews suggest that NMs take on multidimensional leadership behaviors that consist of both enabling and enforcing roles to generate contexts supportive of implementation. In contrast, no studies were found on the role of ANMs in implementation despite their day-to-day involvement in the operations and care processes in a care setting.

Nonmanagerial nursing leaders at the POC also have responsibilities for engaging in processes that aim to optimize the health system, including research, leadership, and education [12]. However, in contrast to research on the role and behaviors enacted by NMs, no studies were found that explicitly explored the IL behaviors of these other types of nurse leaders. Rather, formal nonmanagerial leaders including NPDEs and APNs such as clinical nurse specialists and nurse practitioners, have typically been described in the literature as internal facilitators [13]. Facilitators use strategies that exert social influence to support behavior change including encouragement, role modeling, information sharing, peer-to-peer coaching, delivering formal and informal education, practice surveillance, demonstrating a commitment to the goal, and planning and goal setting among others [14-16]. Similarly, informal nurse leaders can take on champion roles in implementation, and like internal facilitators use a wide range of strategies that are congruent with their professional knowledge, skills, and practice expectations to support change [14]. While it appears that both leadership and facilitation are needed to support implementation, there is considerable overlap in these processes. In addition, it is unclear how the adoption of either facilitation or leadership roles and behaviors is influenced by the type of nursing leader position held, suggesting an important area for clarification and exploration.

Nursing leaders at the POC level may need better training to take on their leadership roles, and in particular to develop strategic IL behaviors to support implementation [17]. In a study by Lunden et al [18], nursing staff perceived weakness in the way their managers enabled implementation, particularly around providing resources, mitigating obstacles to implementation, and engaging in discussions about EBPs. When measuring IL-specific behaviors, Shuman et al [2] found NMs in acute care units only exhibited these behaviors moderately and suggested that interventions to strengthen IL were needed. However, it is unknown whether formal nonmanagerial nurse leaders also have similar performance gaps enacting IL. Some studies were found that described the development and feasibility testing of IL interventions as an implementation strategy and these were directed mainly at POC NMs to support the adoption of a clinical practice guideline [19,20]. A greater understanding of nursing leaders’ behaviors and their interactions is needed to inform how these IL interventions could be adapted to address a team of diverse nursing leaders at the POC involved in implementation.

Nursing leadership to support implementation at the POC resonates with collective leadership models such as shared leadership, which moves away from a perspective of leadership that resides within an individual with formal authority and influencing power [21]. In contrast, shared leadership is a dynamic and collaborative relational process where influence is distributed among a number of networked individuals to achieve team or organizational goals [22,23]. A shared leadership model is viewed as a strength for teams completing complex tasks such as change and improvement in health care contexts and acts as a facilitator for organizations engaged in large-scale transformative change [24,25].

Research exploring the relational processes among nursing leaders that contributed to the successful implementation of EBPs is very limited. In a Canadian study evaluating the feasibility of an IL educational intervention, formal and informal leaders assigned greater value to generating collective versus individual action plans to implement a fall prevention guideline in residential facilities [19]. In another Canadian study, Fleiszer et al [26] described long-term and routine use of clinical practice guidelines in acute care units where the POC nurse leadership team worked cohesively and collaboratively to integrate implementation and sustainability strategies. However, these studies only alluded to the notion of shared leadership in implementation, as an attribute of effective leadership in nursing. In a study examining relational processes among NM and clinical leader dyads to implement urinary incontinence guidelines, van der Zijpp et al [27] concluded that implementation progressed when these leaders were “in sync.” Further, this study pointed to the influence of the organizational culture on POC leaders, suggesting that leaders were reluctant to engage in their implementation role due to specific context factors including the challenges of managing competing priorities, hierarchical leadership structures, and the emphasis on top-down compliance to practice standards [27]. This indicates that more research is needed to explore factors influencing the enactment of IL by different formal and informal nurse leaders as a collective activity at the POC.

In summary, the conceptualization of IL is at odds with nursing leadership at the POC, where multiple leaders in distinct roles are called to lead the implementation of EBPs. Similarly, empirical work to date related to IL in nursing has focused predominately on the NM role, whereas how POC nursing leadership consisting of various formal and informal leaders is enacted to support the implementation of EBPs has been relatively unexplored. This represents an important knowledge gap in the field of implementation science and an area of conceptual evolution for IL. Moreover, how these various leaders work together to support implementation and what influences these processes has not been explicitly researched in depth.

## Methods

### Research Questions

The central research question is as follows: How is IL shared among members of the POC nursing leadership team in inpatient hospital units? The subquestions are as follows: (1) What IL behaviors are enacted and shared by POC formal and informal leaders? (2) What social processes enable shared IL by formal and informal POC leaders? and (3) What factors influence shared IL among the POC nursing leadership team?

### Design

We will use a collective case study approach within the constructivist paradigm to answer the research questions [28]. Collective case study is an approach to inquiry where the researcher explores the particularities of multiple, contemporary, and real-life bounded systems (the cases) in order to generate an in-depth understanding of a social phenomenon [29]. In this study, the cases will include POC nursing leadership teams on 2 inpatient hospital units at a university health network where the implementation of an EBP was successful. In line with the research questions for this study, a case study answers “how” questions by disentangling complex relationships among various factors and processes, which emerge as a function of context both within and external to the case [29]. Case studies are also useful in exploring phenomena that have not been extensively researched, such as shared IL [30]. The emphasis on context distinguishes case studies from other qualitative approaches and promotes the use of multiple data sources and collection methods to develop a holistic understanding of the phenomenon [31]. The selection of multiple cases can generate a greater and more nuanced understanding of how effective shared IL is manifested in different environments, offering the potential to develop an explanatory framework of IL in POC nursing leadership teams [28].

### Constructivist Paradigm

Applied widely across different disciplines, the methodological choices in the design of case studies can be approached from different ontological and epistemological orientations [32,33]. Constructivism, based on a relativist ontology, ascribes to the belief that knowledge of the world is socially constructed [34]. A constructivist orientation to the case study assumes that understanding of a phenomenon such as shared IL in the social world occurs through the perspectives of individuals experiencing it, their shared meanings, and the interactive processes within a given context [35]. For this study, we understand leadership, as constructed through the interactions between grouped individuals, influenced by context and not merely assigned to formalized roles. The focus of this inquiry will be to explore these leaders’ IL behaviors and the social processes that support shared IL from the perspectives and collective experiences of POC nurse leaders in inpatient hospital units. To meet the epistemic commitment to constructivism, the theorizations by Stake [34] characterizing case study as a holistic, empirical, interpretive, and empathic research approach will shape our methodological decisions, the role of the researcher in data coconstruction and interpretation, notions of data validation (eg, triangulation), and implications for knowledge generation.

### Conceptual Framework

**Figure 1:**
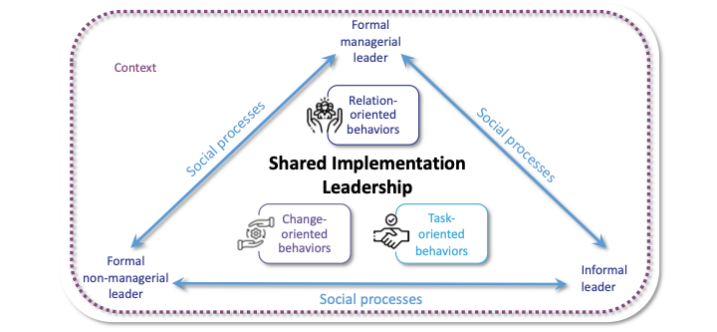
Conceptual framework of shared implementation leadership.

We developed a conceptual framework to guide the selection of key informants and frame the analytical and interpretive focus to explore shared IL among POC nursing leadership team members (Figure 1). A description of the terms can be found in Textbox 1.

This study will build on the dimensions of IL behaviors described in the Ottawa Model of Implementation Leadership, an empirically informed theoretical model in nursing developed over a decade of research [7]. The Ottawa Model of Implementation Leadership describes three meta-categories of leader behaviors to facilitate implementation and support the adoption of EBPs at the POC: (1) relations-oriented behaviors that include supporting, developing, recognizing, and empowering behaviors; (2) change-oriented behaviors that include advocating and envisioning change, encouraging innovation, and facilitating collective learning; and (3) task-oriented behaviors that involve planning, clarifying, monitoring, and problem-solving behaviors. This model is featured in the center of the conceptual framework to describe the specific IL behaviors that are enacted by the different leaders at the POC. Positioned around the IL behaviors are the formal and informal nursing leaders, to integrate the notion that IL behaviors are distributed among a diversity of leaders at the POC. The different leaders are linked to highlight their grouping as a leadership team and social processes where shared IL emerges. Social processes are the patterns of social interactions, that is, the actions and activities occurring over time, and are embedded in a dynamic context, that shapes how IL is shared and the activities across phases of an implementation project [36].

**Description of terms (conceptual framework).**

**Implementation leadership**

- Effective leadership behaviors facilitate the implementation and adoption of evidence-based practices at the point of care.
- Meta-categories of leadership behaviors described in the Ottawa Model of Implementation Leadership [7]:
  Relation-oriented behaviors: Supporting, developing skills, and recognizing others and their contributions to increasing trust and cooperation.
  Change-oriented behaviors: Integrating a vision, demonstrating commitment, building coalitions to support change, and creating a sense of need.
  Task-oriented behaviors: Planning, clarifying roles, monitoring performance, and efficiently using resources.

**Leaders**

- Formal managerial: Individuals with appointed administrative and management roles with explicit and legitimate authority on the unit (eg, nurse manager and assistant nurse manager).
- Formal nonmanagerial: Individuals with formally recognized clinical leadership and professional development roles in the organization (eg, advanced practice nurses and nursing professional development educators).
- Informal: Individuals with a staff nursing position on the unit are viewed among peers and formal leadership groups as credible. Formalized in the role of nursing champion in the context of an implementation project.

**Social processes**

- Patterns of social interaction within the team (ie, the individual and collective actions and activities) over time where point of care leaders mutually influence each other for shared implementation leadership to emerge [36].

**Context**

- The structural, political, cultural, and historical environments of the inpatient unit and health care organization shape features of the social processes.

### Sampling and Recruitment

#### Cases

Stake [34] defines a case as a “bounded system” from which much can be learned. With multiple cases, each case is unique and studied in detail, but it is the phenomenon expressed within each case and across cases that is of interest [28]. We will select 2 instrumental and bounded cases from a large bilingual teaching health network in Montreal, Canada to gain a broad appreciation of shared IL, the phenomenon of interest (or “quintain”) in this study [28]. The case will be operationally defined as an inpatient unit in which the POC nursing leadership team has successfully implemented a specified EBP. Inpatient units are socially distinct microsystems that interact with external features, such as the larger organizational hospital context [37,38]. Therefore, inpatient units are considered individual cases, but embedded within the broader clinical and administrative organization and health care contexts. The cases will be bounded as follows: (1) spatially by the physical location, (2) purposefully, by the local clinical mission of the unit, where members of the POC nursing leadership team are assigned responsibility for and have supported the successful implementation of a specified EBP, and (3) temporally by the phases and timeline boundaries of the implementation project.

#### Case Selection

We will select individual cases based on their relevance to the quintain, the diversity of contexts, and access [28]. To identify potential cases, we will seek advisement from the senior leaders at the health network who are responsible for coordinating EBP project implementations and knowledgeable of implementation outcomes of potential cases. The first case (case A) will be purposively selected and based on predetermined criteria: (1) The successful implementation of the EBP project on the unit within the previous 6 months and senior NMs perception of a “strong” nursing leadership team on the unit, (2) the presence of diverse leaders at the POC, and (3) the availability of the nursing leadership team on the unit to participate in the study. In light of emerging findings as the study unfolds, we will use maximum variation sampling to identify the second case (case B) in consideration of different contextual elements influencing the case (eg, nursing leadership team factors such as composition and tenure, clinical mission, hospital site, and implementation project) [39].

#### Data Sources

A variety of data sources including key informants and documents will provide breadth and depth to understanding the quintain. We will purposively select key informants for their perspective of firsthand experiences in sharing IL including POC nursing leadership team members consisting of formal managerial and nonmanagerial leaders (ie, the NM, ANMs, NPDE, or APNs) who are assigned responsibility to the inpatient unit and were present during the implementation of the specified EBP; and informal leaders (ie, staff nurse who worked on the unit and were selected as a champion for the specific EBP implementation project). Through purposive and snowball sampling, we will also recruit key informants from the unit and organizational level, who interacted with or observed POC nurse leaders during the implementation, had a role in influencing the process of shared IL, or can offer a perspective on the contextual influences on shared IL (eg, interdisciplinary team members, staff nurses, and senior-level nursing administrators) [40]. Key informants are considered within the boundaries of the case if they are directly involved in the social processes where shared IL emerges. On the other hand, they will be considered external to the case if they are observers or influencers of the process. Participants will be identified iteratively as the study unfolds with the aim of sampling as many key informants as possible to acquire saturation and depth in understanding the quintain as manifested for each case [41]. Recruitment of POC nursing leadership team members will be completed first and facilitated by senior leaders in the health network. The total number of anticipated key informants recruited will range from 10 to 19 per case.

The documents collected for this study will provide background and historical information and will serve as a proxy for observations [42]. The content and information contained within documents may be useful in describing the quintain over time and can extend, clarify, support, or contradict the perspectives of the key informants. Documents may also provide insight into leadership roles in implementation-specific events, point to instances and processes of leadership being negotiated and distributed, and establish formal expectations for POC leadership in implementation [29]. These insights will inform ongoing data collection by including new interview questions and identifying other documents to gather [42]. Moreover, the documents themselves can be viewed as serving a specific function within the setting [43]. For example, documents created by the setting and using a particular form and structure can give insight into the context of unit and organizational values influencing the social processes among leaders. Examples of documentary sources for this study can include written organizational documents (eg, job descriptions, project charters, protocols, meeting notes, and annual reports), electronic material (eg, email messages and presentations), and physical objects (eg, “war rooms” or “quality boards”, bulletin/whiteboards, and unit layout schematics).

### Data Generation

#### Interviews

We will conduct focus groups with the POC nursing leadership team first. As a pre-existing socially constructed group, participants will be able to draw on their shared experiences and individual group members’ recollections to construct a deeper understanding of the quintain [44]. Data collected from this facilitated discussion can highlight whether accounts and experiences are perceived similarly or differently across team members [45]. Due to the retrospective nature of the case, a facilitated discussion between team members may offer an approximation of their natural group interactions and provide insight into the nature of their relationships (eg, power differentials and conformity) and social processes (eg, reaching consensus and groupthink) [46]. As group interactions are largely nonverbal, observations of group dynamics, including forms of communication, patterns of discussion, group norms, and body language will be collected in the form of descriptive field notes [45-47].

We will conduct individual interviews with key informants for each case to acquire a breadth of perspectives to shape our understanding of shared IL and factors that influence the social processes of shared IL. Complementary to the focus group, individual interviews will yield granular accounts of the case, including greater depth into issues raised during the focus groups, perceptions of shared IL processes and influence, and to check assertions about the group dynamic [48]. Individual interviews with other key informants will clarify their interactions with members of the POC nursing leadership team, and their perceptions of how the POC nursing leadership team supported implementation and influencing factors. The principle of data saturation will guide the total number of interviews conducted for each case [41]. Minimally, 1 focus group interview (possibly over 2 sessions) and 1 individual interview with key informants (n=10-19) will be conducted per case. Additional interviews (focus group and individual) will likely be needed and will serve to validate the analysis (ie, member checking), discuss interview transcripts, clarify ideas, or explore emerging notions gleaned from other data [49].

For both focus group and individual interviews, we will offer face-to-face, in-person or digital interviews, at the convenience of the participants. Interviews will be audio-recorded to capture verbal content data. Prior to the interviews, all participants will complete a sociodemographic questionnaire (Multimedia Appendix 1), which will enable the reporting of details of the key informants in the case description. Interviews will be semistructured to foster an emic approach in line with the constructivist view. We developed an interview guide (Multimedia Appendix 2), with open-ended questions linked to key concepts in the conceptual framework, to explore topic areas aligned with the focus of inquiry while responding to emerging issues emanating from the participants’ experiences of the case [37]. The main questions in the interviews will relate to a description of the EBP project on the unit, the interviewee’s involvement in the EBP implementation, IL behaviors that were distributed or collectively enacted by POC nursing leaders throughout the implementation phases, activities that enabled the POC nursing leadership team to share IL, and the perception of factors related to the context that facilitated or constrained how POC leaders shared IL. In addition, the guide incorporates elements aimed at developing rapport with participants such as broad open-ended questions, with prompts and probes to encourage information sharing and to clarify points [47]. The interview guide concludes with ending questions to promote member checking by summarizing and clarifying what was discussed and asking participants to refer to other sources of information [30]. Throughout the course of inquiry, the interview guide may be adapted in light of emerging ideas and notions from other sources (eg, documents) [47]. Interview guides will be piloted for clarity and comprehension prior to the first interview.

We will identify documents in relation to each case by searching organizational papers and web-based databases, and by direct request of key informants. In addition, SAC will make unit site visits (brief 30-minute tours of the units guided by a leader on the unit to identify other relevant documents) and capture observations about the case through researcher-generated field notes. Identified documents will be scanned, digitally photographed, saved electronically in a secure study database, and logged. A document intake form will be used to log the characteristics of the document (eg, when, how, and from whom the documents were retrieved), the authenticity of the document, and key themes from the document that relate to the study questions (Multimedia Appendix 3 [40]).

We will generate several types of field notes throughout the course of data collection to accurately describe the research process and achieve an in-depth and credible interpretation of the findings [50,51]. Jottings of ideas, impressions, and brief details of observations will be noted sporadically on a paper notepad to serve as a memory jogger. A daily log of anticipated and actual data collection activities, activities that are being considered, and brief profiles of key informants will be kept electronically. Descriptive observational field notes will be written on separate electronic documents immediately following the case selection meetings, interviews, and unit site visits. Methodological field notes will capture reflections on the techniques and methods used, their perceived success, and adaptations in future data collection. Analytic memos will be used to develop ideas and interpretations of the data collected. Finally, a personal reflexive journal will be kept recording thoughts, feelings, and biases noticed throughout the research process.

#### Data Analysis

A 2-phase data analysis process will be conducted to ultimately deepen our understanding of the quintain: Within- and cross-case analysis [28]. A within-case analysis will generate an in-depth description of each case, the context and case-based themes, and will occur simultaneously with data collection so that emerging data can iteratively inform the selection of the second case, the pursuit of additional key informant perspectives, and direct lines of inquiry [29]. The Framework Method for the analysis of qualitative data will guide a structured, interconnected, and transparent flow of activities for an in-depth thematic analysis of the data and is aligned with the phases of case analysis described by Stake [34] (ie, case description, categorical aggregation, pattern recognition, and naturalistic generalization) [52]. Similarly, the Framework Method integrates inductive and deductive approaches to analysis, capitalizing on a balance of etic and emic constructions of the case. This approach generates matrix outputs where a large amount of diverse textual data (eg, interviews, documents, and field notes) are systematically reduced, summarized, and displayed facilitating a team approach to analysis [53,54].

The Framework Method moves through five stages: (1) familiarization with the data to form hunches about emerging issues or concepts that correspond with the conceptual framework for each case, (2) developing and iterative refinement of a thematic framework through deductive and inductive coding from a set of a priori codes and subcodes derived from the conceptual framework, (3) indexing the remaining data with the thematic framework, (4) charting of the indexed data into a matrix to allow for data from the entire case to be viewed, and (5) mapping of patterns among categories and interpretation of the data. This final step will be carried through a back-and-forth process between data categories, raw data, and analytic memos toward understanding the case and establishing relationships between case themes and the context.

The case reports generated from the within-case analyses will build toward a cross-case analysis, which aims to identify commonalities and differences of themes across the corpus of cases, and how these are influenced by the variations in contexts to make assertions about the quintain [28]. Assertions constructed about the shared IL may support or suggest modifications to the conceptual framework initially proposed for the study. An updated framework will inform the conceptual structure for the final cross-case report and will be examined against existing research and theory.

### Ethical Considerations

The study protocol received research ethics approval (2022-8408) on February 24, 2022, and renewal on February 21, 2023. The study will undergo a research ethics review. There is minimal risk of harm to participants in this study. Potential participants will be informed about the study (eg, study purpose, nature of their involvement, potential risks, and benefits) and will be required to provide written consent prior to embarking on the study. The right to refuse to participate and withdraw at any time during the study without consequence will be explained. The anonymity of study participants will be ensured by identifying transcripts with a numerical code, with the code key kept separately; storage of data files in secured physical and electronic locations; and limited access to data files by the research team [30]. Due to the nature of case study research and its use of thick description to report on the uniqueness of the case, there is a risk of deductive disclosure especially as the POC leaders are known to all staff [55]. In addition, confidentiality will be impossible to maintain for group interviews. Therefore, additional safeguards will be put into place to ensure confidentiality and build rapport with participants. These include (1) a discussion with interviewees following data collection about the anticipated audiences for dissemination of the study results, (2) offering participants the opportunity to review interview transcripts, (3) consulting with participants on how to treat text that may be identifying in nature for the purposes of data analysis and dissemination, and (4) in group interview circumstances, reminding participants not to share any details of the discussion outside of the group context [55]. As interviews with participants will be held during work hours, compensation will not be provided.

### Reflexivity and Rigor

SAC will conduct this study to partially fulfill the requirements toward a PhD in nursing degree. She will conduct this study as a trainee with the research institute in the setting and will be partially subsidized by the nursing directorate. SAC is a registered nurse and has worked in the study setting as a clinical nurse specialist and then as an advisor for EBP. As a function of these roles across different sites within the setting, SAC believes she has earned credibility and trust from colleagues, particularly nurses in leadership roles. SAC maintains a strong connection with senior leaders as well as with other colleagues. As a research trainee and recent previous employee with the setting, SAC believes to be positioned as an insider, due to the “lived familiarity” with the participants in the research and a priori knowledge of the setting and potentially of the cases as well [56]. As an insider, she collaborated with senior leaders in the conceptualization of the study, to strengthen the feasibility and alignment of the study plan with departmental expectations. SAC’s insider status also raises questions about the implications of power with respect to data collection, disclosure of confidential knowledge and perspectives, and reporting that may negatively influence the site, participants, and herself [29]. In addition to the strategies mentioned above, SAC will strive to be transparent in interactions with the individuals in her role as a researcher and demonstrate openness to address any concerns in the field [29].

Integrated into the design of this study proposal are strategies that enhance the trustworthiness of a naturalistic inquiry and meet the criteria for a good case study report [34,35]. The strategies encompass researcher behaviors, inquiry, and reporting processes to promote confidence in the findings presented on shared IL (credibility criteria), that accepted research practices were followed in exploring shared IL (dependability criteria), that the findings are grounded in an emic perspective (confirmability criteria) and that there is sufficient contextual information presented in the reporting of this study on shared IL for readers to deliberate over the findings for applicability in other contexts (transferability) [57]. Table 1 details the compendium of strategies planned for each quality criterion.

**Table 1:** Methodological rigor criteria, definitions, and strategies.

Rigor criteria [58]	Definitions [57]	Strategies to enhance rigor	Criteria described by Stake [34] for a good case study report (adapted)
Credibility	The extent to which the reader has “confidence that [the researchers] have accurately recorded the phenomenon under scrutiny” [57]	Adequate engagement in the field to generate data [40] Triangulation to promote variation in views and ways of constructing data Establishing rapport in interactions with key informants [57,59] Member checking to validate the accuracy and interpretation of data [34] Debriefing sessions with research team to discuss and challenge data construction Transparency in data construction by engaging in reflective commentary [57]	Data sources are well chosen and in sufficient numbers. Observations and interpretations appear to be triangulated. Sound assertions appear to be made, neither over- nor under-interpreting. Individuals were not put at risk during the inquiry.
Transferability	The extent to which the reader has been provided sufficient contextual information by the researchers to apply the findings elsewhere	Thick case description [57] Maximum variation [40]	Case adequately defined. Reader is provided with some vicarious experience (sense of story). Adequate attention paid to various contexts.
Dependability	The extent to which the reader can assess that the proper research practices have been followed	In-depth methodological description [60]	Final report is easy to read and edited well. Research questions and themes developed in a serious and scholarly way.
Confirmability	The extent to which the reader can determine that the findings are based on an emic perspective, “rather than the characteristics and preferences of the researcher” [57]	Researcher reflexivity [57] Demonstration of an audit trail [44,57]	Findings have a conceptual structure (ie, themes). Quotations are used effectively. Tables and figures are used effectively. Sufficient raw data are presented. The role of the researcher is apparent. Empathy is shown. Researcher’s personal intentions are examined

## Results

Data collection began in June 2022, and we have recruited 2 inpatient hospital units and 24 participants to date. We experienced several challenges locating and recruiting key informants (ie, no longer employed at the hospital) or delays in scheduling focus group interviews with nursing leadership teams and individual interviews with key informants due to high levels of workload during the COVID-19 pandemic. We amended the study protocol (approval received on July 18, 2022), with the research ethics board to be able to recruit employees no longer working at the hospital. We anticipate data collection to be completed by January 2024 and findings to be published in a peer-reviewed journal by late 2024.

## Discussion

### Significance and Anticipated Contributions

Over, the next 10 years, Canada has committed to advancing the science of knowledge mobilization to generate more capable, effective, and humane health systems and to improve the health of individuals and populations [61]. The aims of this study address this priority by exploring more deeply how nursing leadership facilitates the successful implementation of EBPs to ensure high quality care delivery. Further, as nurses represent the largest regulated health professional group in health systems worldwide and are recognized as key drivers in care quality, the findings of this study will be an important contribution to this goal [62,63]. This study will answer calls for further research investigating how leadership as a complex contextual factor interacts with the implementation process and will add to the growing body of knowledge on POC leader roles in implementation [64]. By expanding the conceptual understanding of IL as behaviors enacted by a single managerial leader, we will be able to articulate in detail the specific contributions of formal and informal nurse leaders at the POC fostering implementation climates. This study will be the first one to hold a relational lens to IL, explore the social processes of an intradisciplinary team at the POC, and demystify the characteristics of effective leadership teams in facilitating evidence-based changes in nursing.

The knowledge generated from study findings can inform the development of tailored implementation strategies that seek to bolster nursing leadership at the POC, such as how to adapt interventions aimed at single POC managers for POC nursing leadership teams. Other nursing scholars in implementation are similarly exploring ways to “prepare context” that easily adapts to regular and ongoing implementation efforts and poses “fewer barriers to change” [65]. By equipping nurse leaders to engage staff and support consistent success in implementation efforts, we may lessen the cost of failed implementation efforts and generate healthier work environments for nurses [66]. Nursing leadership is distinguished from other health care professionals through its multiple and diverse leadership roles at the POC, yet little is known about their specific contributions in implementation, specifically ANMs. The findings may help to inform nursing administrators about what organizational supports are needed to strengthen effective IL at the POC and to facilitate the development of emerging nurse leaders. In addition, during the COVID-19 pandemic, NMs reported that their roles and responsibilities had expanded and sometimes changed altogether with little or no preparation and training [67]. NMs may be increasing their reliance on other POC leaders to distribute specific leadership tasks in order to support implementation and the quality of care in their units. This study is timely and can offer much-needed clarity for policymakers on the present-day contribution of formal and informal roles in nursing to the provision of quality care.

### Limitations

One of the limitations of this study is the generalizability of the findings to other inpatient hospital units or health care settings, due to the idiosyncratic nature of the qualitative case study approach and exploration of 2 cases in 1 setting [29]. However, the in-depth focus on the particular and unique aspects of the cases will illuminate the complexity of leadership supporting implementation in nursing and the importance of context in shaping its manifestation [34]. There may be concerns about social desirability bias limiting what POC nursing leaders discuss and disclose during interviews, given the legitimate power relationships across leader types (ie, manager and subordinate relationship) and their ongoing working relationship [68]. Yet, it is the analysis of this social context (eg, group norms) that is of interest to this study. Increases in workload, shifted priorities due to the ongoing COVID-19 pandemic and the nursing workforce shortage in the wake of the acute pandemic phase presented challenges with recruitment and availability of POC nurse leaders, and other key informants in the health setting. We are offering digital interviews to improve the likelihood of participation by teams and individuals. POC nursing leaders may be accustomed to communicating with each other digitally because of the pandemic and therefore may approximate their interactions in the natural setting.

### Conclusion

Implementation of EBPs drives and sustains high-quality nursing practice, where effective nursing leadership at the POC is integral to its success. This protocol for a collective case study aims to conceptualize IL through a shared leadership lens in nursing. The study findings will have broad implications for ongoing research in the fields of implementation science and nursing leadership; leadership practice by providing a model for leadership that enables successful implementation; developing leadership education competencies for current and emerging nursing leaders; and informing structural and cultural policy changes that support nursing leader roles in the health system.

## Appendix

Multimedia Appendix 1 Sociodemographic questionnaire.

Multimedia Appendix 2 Semistructured interview guide.

Multimedia Appendix 3 Document intake form.

Multimedia Appendix 4 Peer-review report by the McGill University Doctoral Comprehensive Examination Committee.

## Abbreviations

ANM: assistant nurse manager
APN: advanced practice nurse
EBP: evidence-based practice
IL: implementation leadership
NM: nurse manager
NPDE: nursing professional development educator
POC: point of care

## Data Availability

The data sets generated or analyzed during this study will not be publicly available due to the small sample size and risk of participant identification in this study, and in adherence to the requirements of confidentiality in line with the present ethics permission.
