# Supplementary material for: Exploring Shared Implementation Leadership of Point of Care Nursing Leadership Teams on Inpatient Hospital Units: Protocol for a Collective Case Study
Source: JMIR Res Protoc. 2024 Feb 19;13:e54681. doi: 10.2196/54681 (PMC10912983; doi:10.2196/54681)
Supplement: Multimedia Appendix 1 [file resprot_v13i1e54681_app1.docx]

Multimedia File 1

Participant Sociodemographic Information Form (English)

| Participant ID: |  | Date: |  |
| --- | --- | --- | --- |

Instructions:

Please answer the following questions pertaining to you, your professional and work experience. Your answers will be combined with others to describe the general characteristics of the group of participants in this study.

|  | What is your gender? | - Woman - Man - X (Transgender, non-binary, two-spirit) - Other: ___________________________ - Prefer not to answer |
| --- | --- | --- |
|  | What is your age? | ___________ years   - Prefer not to answer |
|  | What is your profession? | - Nurse - Other: ____________________________ |
|  | What is your highest level of education? | - CEGEP - Bachelors - Masters - PhD - Other: ____________________________ |
|  | How long have you worked in your profession? | _______________ years and _____________ months |
|  | How long have you worked at the MUHC? | _______________ years and _____________ months |
|  | What is your current job title? | ____________________________________ |
|  | What is your current employment status? | - Full-time - Part-time: _________days worked per week - Other: _________________________________________ |
|  | How long have you worked in your current job title? | _______________ years and _____________ months |
|  | How long have you worked on [unit A/B]? | _______________ years and _____________ months |
|  | Have you participated in any formal training or professional development on the implementation evidence-based practices? | - No - Yes. Please briefly describe when and what training you received: |
|  | Have you participated in any formal leadership training? | - No - Yes. Please briefly describe when and what training you received: |

*End of questions*

**Thank you for your participation!**

[version number in footer]
