# Supplementary material for: Exploring Shared Implementation Leadership of Point of Care Nursing Leadership Teams on Inpatient Hospital Units: Protocol for a Collective Case Study
Source: JMIR Res Protoc. 2024 Feb 19;13:e54681. doi: 10.2196/54681 (PMC10912983; doi:10.2196/54681)
Supplement: Multimedia Appendix 2 [file resprot_v13i1e54681_app2.docx]

Multimedia Appendix 2

Draft of Semi-Structured Interview Guide

For each interview:

| **Set up** |
| --- |
| - Configure space - Connect and test audio-recording device - Double check that consent form(s) are signed |
| **Introduction** |
| - Introduce research team   - Discuss facilitator and research assistant roles (when applicable) - Description of the project and purpose of the interview   - Review main objectives of the study   - Specify focus of the discussion is bounded by the [EBP project] implemented on the unit   - Encourage questions about the study - Assurances of participant rights in the research study   - Emphasize anonymity in data collection processes and participant feedback regarding published reports   - Reiterate restricted access to the raw data collected and data analysis   - Remind participant(s) that they may choose to withdraw at any time   - Encourage questions - Outline interview format   - Anticipate interview will last approximately 30-60 minutes (possibly over 2 sessions for the focus group)   - Remind participant(s) of audio recording (audio-visual in case of virtual interview)   - Encourage participant(s) to ask for clarification when needed and to answer questions to the best of their ability (i.e., “no right or wrong answers”)   - Ask participants to speak freely, ask each other questions to stimulate the discussion   - Ask participant(s) to avoid talking over another, for ease of transcription. Instruct on use of “hands up function” for virtual interview. |
| **Examples of prompts to encourage further discussion, elaboration, clarification, transition** |
| - “That is interesting, please continue/elaborate”, “Go on”, “tell me more about…” - “What happened when…”, “What did you do when…? “Why/why not?” “How did this come about?” “What makes you say that?” - “What is an example of…?” “Can you think of another example?” “When was this not the case?” “Describe what happened next” - “What do others think about…?” “What are other’s perspectives on this?” “How do others see this?” - “We’ve been talking about…, Could we now move on to…” |
| **Wrap up** |
| - “Let me summarize the main points of the discussion…is this an adequate summary?” “Did I miss anything?” “How would you summarize our discussion?” - “Who else should I speak to learn more about…?” - “What documents may be important for me to review to further understand/illustrate how…the implementation of [EBP project]?” - If not complete, ask participants to complete **Participant Sociodemographic Information Form** |

Question guide by interview and informant type:

| **Focus Group: POC Nursing Leadership Team^1^** |
| --- |
| *Part 1: Describing leadership roles and behaviors manifested by the project* |
| - Please introduce yourself and your role in the organization/on the unit - Tell me briefly about the leadership team. How long have you worked together on the unit? - Tell me about the implementation of [EBP project] - How did you (each role) support the implementation of this project? What did you do?   - Anything else to add about how [this leader] supported the implementation of [EBP project]? |
| *Part II: Exploring perceptions about how leadership was shared during the project* |
| - Which of these described behaviors were shared among different leaders of the team?   - How did “sharing” happen?   - How were behaviors divided?   - How did you decide who does what? - What were the strengths of “sharing” leadership to support this project? - What were some of the challenges when “sharing” leadership to support this project? - How did you, as a team, overcome a particular challenge during this project? - You are recognized by [senior leaders/administrators/others in the organization] as a strong performing leadership team. Why do you think so? - How did your leadership team contribute to the success of the project? |
| *Part III: Exploring influencing factors on sharing of leadership* |
| - What do you think about the notion of sharing leadership? - What do you think enabled how you worked together to support the implementation of the project?   - What hindered it? - What are the strengths of your team in support of this project? What areas need improvement based on your experience during this project? |
| **Individual Interview: POC Nursing leaders** |
| *Interviews will be open-ended and will build on the focus group findings to:*   - *Describe granular accounts of the case* - *Explore issues constructed from the focus group discussion in greater depth* - *Check assertions about group dynamics interpreted in the focus group* |
| **Individual Interviews: Other Key Informants** |
| - Please introduce yourself and your role in the organization/on the unit - How were you involved in the implementation of [EBP project]? - Who on the (nursing leadership team) did you work with on the unit for this project?   - What was the nature of your collaboration/work together in this project? - What aspects of nursing leadership supported the implementation of the [EBP project] on the unit? - What influenced how nursing leaders on the unit contributed to the success of the [EBP project] on the unit? |

^1^ *The focus group guide will be the same for each case*

*Note: Individual interview questions will evolve iteratively from the focus group and in consideration of other data sources.*
