# Supplementary material for: Exploring Shared Implementation Leadership of Point of Care Nursing Leadership Teams on Inpatient Hospital Units: Protocol for a Collective Case Study
Source: JMIR Res Protoc. 2024 Feb 19;13:e54681. doi: 10.2196/54681 (PMC10912983; doi:10.2196/54681)
Supplement: Multimedia Appendix 3 [file resprot_v13i1e54681_app3.docx]

Multimedia Appendix 3

Draft of Document Intake Form

| **Item** | **Details** |
| --- | --- |
| *Document title* |  |
| *Document date* |  |
| *Type of document (e.g., email, protocol, photograph)* |  |
| *Obtained from (e.g., person, database)* |  |
| *Document history (how did it come about?)* |  |
| *Authenticity and completeness of the document (Does it appear genuine? Is anything missing?)* |  |
| *Purpose of the document (Why was it produced?)* |  |
| *Main and collaborating authors, sponsors* |  |
| *Intended audience(s)* |  |
| *Information (content) presented or discussed* |  |
| *Source of information presented* |  |
| *Other documents referenced* |  |
| *Themes that are relevant to the study questions* |  |
| *Other comments* |  |

Adapted from [Merriam and Tisdell (2016)](#_ENREF_103)
