# Supplementary material for: Exploring Shared Implementation Leadership of Point of Care Nursing Leadership Teams on Inpatient Hospital Units: Protocol for a Collective Case Study
Source: JMIR Res Protoc. 2024 Feb 19;13:e54681. doi: 10.2196/54681 (PMC10912983; doi:10.2196/54681)
Supplement: Multimedia Appendix 4 [file resprot_v13i1e54681_app4.pdf]

McGill | Ingram School of Nursing | École des sciences infirmières Ingram

DOCTORAL COMPREHENSIVE EXAMINATION – EVALUATION FORM A – WRITTEN COMPONENT

Summary report of the CHAIR

Student: Sonia Angela Castiglione, RN, MScA, PhD Student

Title of Proposal: Exploring Shared Implementation Leadership of Point of Care Nursing Leadership Teams on Inpatient Hospital Units: A Collective Case Study

Members of the Comprehensive Examining Committee:

1 - Anita Gagnon (Chair)

2 – Sonia Semenic

3 – Mélanie Lavoie-Tremblay

4 – Isabelle Brault (External Examiner)

EVALUATION OF WRITTEN PROPOSAL:

1. INTRODUCTION/BACKGROUND: Relevant literature is concisely summarized. Gaps in current knowledge and/or limitations of previous research are clearly identified and provide a rationale for the proposed study. The theoretical/ conceptual underpinnings of the study are well articulated and justified.

☐**Pass** ☒**Pass with Revisions** ☐**Fail**

Comments for revisions:
Clearly written with relevant appendices. A few minor edits are needed. The case for why "…how formal and informal nurse leaders at the POC share IL [...] represents an important knowledge gap" (p. 4 & 14) and that it "…warrants in-depth exploration" (p.4) is not well justified. A strong case is presented that there aren't studies looking at this but a weaker case for why it is important to know.

The POC Leader Roles in Implementation section would benefit from more contextualization of the studies reported - countries, staffing configurations, role of professional licensing bodies and associations, etc. This would allow for a better sense of the transferability of what you'll find in your study.

Comments for revisions: Les sections de l'introduction et le background sont bien présentés. La notion des 'social processes' apparait uniquement dans le cadre conceptuel et mériterait, à mon avis, d'être mieux défini dans la section du background de l'édude. À discuter. Quel est le nom du cadre conceptuel? À identifier.

2. PURPOSE / STUDY QUESTIONS: Study purpose is clearly stated after the literature review and flows logically from the critical review of the literature. Study questions are clear and coherent with the literature review and study purpose.

☐**Pass** ☒**Pass with Revisions** ☐**Fail**

Comments for revisions:
See response to first topic above.

<u>3. METHODS</u>: Design and methods chosen fit with the research question and stated purpose of the study and are clearly outlined in sufficient detail.

☐**Pass** ☒**Pass with Revisions** ☐**Fail**

Comments for revisions:
Minor edits.

Plusieurs auteurs en recherche qualitative sont nommés tout au long de la méthode, il faudrait toutefois identifier plus clairement quel est l'auteur principal, au niveau de la méthodologie, qui guide l'étude

<u>4. SAMPLE / PARTICIPANTS</u>: Specific details and justification for sample size (i.e., power calculation), inclusion/exclusion criteria and process for selecting participants are included and fit with the purpose of the study.

☐**Pass** ☒**Pass with Revisions** ☐**Fail**

Comments for revisions:
Justification for only two units was not given.

The definition of "successful" implementation of EBP has not been given for any of the three potential implementation projects and the difficulty in measuring their implementation is varied across the three. Appendix G defines "successful implementation" as "…the achievement of implementation/project goals". What are they and to what extent have they been met? If recruitment is to begin in January, it would seem that the goals would be known now. Without data to support that the goals have been met, it is unclear how a case can be selected.

<u>PROCEDURES:</u>

<u>5. INSTRUMENTATION / MEASURES / INTERVIEW GUIDES:</u>

For Qualitative studies: Interview guides, observation grids and other data collection tools are broadly and clearly described in text and the specific questions / grids are included in appendices. Questions are simply and clearly stated, ordered in a way that does not bias responses, and flow in an appropriate and logical fashion.

For Quantitative studies: Study indicators, measures and/or questionnaires are fully described along with their psychometric properties (when applicable) in text, and copies included in appendices. Order of administration of measures is explained and justified.

☒**Pass** ☐**Pass with Revisions** ☐**Fail**

Comments for revisions:

6. DATA COLLECTION: Details for data collection procedures are provided such that it is clear what will be done by whom, when and where.

☒**Pass** ☐**Pass with Revisions** ☐**Fail**

Comments for revisions:
Cette section comporte beaucoup d'écriture au 'je/I' à modifier pour les versions ultérieures.

7. DATA ANALYSIS: Data analysis procedures are described in sufficient detail so the steps to analysis are clear and address each of the study objectives/hypotheses appropriately.

☒**Pass** ☐**Pass with Revisions** ☐**Fail**

Comments for revisions:
Proposition: une représentation graphique des différentes phases d'analyse serait intéressante pour lier les phases de Stake aux stages de Gale.

8. METHODOLOGICAL RIGOR: Strategies to ensure methodological rigor are described in sufficient detail.

☒**Pass** ☐**Pass with Revisions** ☐**Fail**

Comments for revisions:

9. LIMITATIONS: Potential limitations of the study are addressed in sufficient detail.

☐**Pass** ☒**Pass with Revisions** ☐**Fail**

Comments for revisions:
More specifics on how you will respond to the potential of social desirability bias is needed.

10. ETHICAL CONSIDERATIONS: Ethical considerations address the issues of confidentiality, voluntary informed consent, withdrawal, risks and benefits of participation, and data storage.

☐**Pass** ☒**Pass with Revisions** ☐**Fail**

Comments for revisions:
Clarity on how coercion (or the perception of coercion) for nurse leaders to participate will avoided is needed given that it seems that confidentiality regarding participation does not seem to be an option.

IMPLICATIONS/CONTRIBUTIONS: The study’s potential implications and/or contributions to nursing theory, practice, education and/or research are well-articulated and relevant.

☐**Pass** ☒**Pass with Revisions** ☐**Fail**

Comments for revisions:
Example of "tailored implementation strategies" (p. 34) and "organizational supports...to strengthen effective leadership" (p. 35) would be key to underline the notion that your study is important and relevant to practise.

WRITING AND FORMAT: Easy to read and follow. Sentences are clearly written. Ideas flow logically. Paragraphs are short and well-defined. Headings are used to organize sections. Paper is carefully edited with few typographical or spelling errors. Proposal body is correctly formatted in APA with a conventional font, size 12-point, 12 characters per inch, with 1-inch margins, double-spaced. All references are properly cited in text and referenced in APA.

☒**Pass** ☐**Pass with Revisions** ☐**Fail**

Comments for revisions:

SUMMARY OF THE EVALUATION OF THE WRITTEN EXAMINATION:

Strengths of the Thesis Proposal:
Overall a strong and well-written proposal.

Le protocole est clairement écrit et les concepts bien définis. Les références sont récentes. Les écarts dans la littérature bien identifiés et le but et questions de recherche clairement développés. Le projet reçoit un soutien important du MUHC ce qui est important pour la faisabilité du projet. L'ensemble des documents nécessaires à l'étude en annexe sont clairement présentés. Félicitations pour la publication de l'article sur l'analyse du concept d'implémentation leadership.

Areas to Improve:
Greater clarity is needed on: the importance of the study to practice; identification of cases based on successful implementation of EBP; and how coercion of nurse leaders to participate will be avoided.

Intégrer un volet sur les 'social processes' dans le background de l'étude. Dans la section de la méthode, mieux statuer l'auteur méthodologique sur lequel s'appuie l'étude. L'échéancier me semble ambitieux pour compléter les deux cas mais pas impossible à réaliser.

FINAL GRADE:

☐**Pass**
☒**Pass with minor revision(s)**
☐**Pass with some major revision(s)**
☐**Fail (must resubmit)**

If Examination is for a Resubmission:

☐**Pass on resubmission**
☐**Fail on resubmission**

SIGNATURES

Chair, Comprehensive Examination Committee: Dr. Anita Gagnon, Associate Dean and Director, ISoN

Signature

Date: 11/16/2021

Director, PhD Program/ or designate:

Signature____

Date:
